# Accurate segmentation algorithm of acoustic neuroma in the cerebellopontine angle based on ACP-TransUNet

**DOI:** 10.3389/fnins.2023.1207149

**Published:** 2023-05-24

**Authors:** Zhuo Zhang, Xiaochen Zhang, Yong Yang, Jieyu Liu, Chenzi Zheng, Hua Bai, Quanfeng Ma

**Affiliations:** ^1^Tianjin Key Laboratory of Optoelectronic Detection Technology and Systems, School of Electronic and Information Engineering, Tiangong University, Tianjin, China; ^2^Tianjin Cerebral Vascular and Neural Degenerative Disease Key Laboratory, Tianjin Huanhu Hospital, Tianjin, China; ^3^School of Computer Science and Technology, Tiangong University, Tianjin, China; ^4^College of Foreign Languages, Nankai University, Tianjin, China

**Keywords:** deep learning, acoustic neuroma, image segmentation, transformer, UNet

## Abstract

Acoustic neuroma is one of the most common tumors in the cerebellopontine angle area. Patients with acoustic neuroma have clinical manifestations of the cerebellopontine angle occupying syndrome, such as tinnitus, hearing impairment and even hearing loss. Acoustic neuromas often grow in the internal auditory canal. Neurosurgeons need to observe the lesion contour with the help of MRI images, which not only takes a lot of time, but also is easily affected by subjective factors. Therefore, the automatic and accurate segmentation of acoustic neuroma in cerebellopontine angle on MRI is of great significance for surgical treatment and expected rehabilitation. In this paper, an automatic segmentation method based on Transformer is proposed, using TransUNet as the core model. As some acoustic neuromas are irregular in shape and grow into the internal auditory canal, larger receptive fields are thus needed to synthesize the features. Therefore, we added Atrous Spatial Pyramid Pooling to CNN, which can obtain a larger receptive field without losing too much resolution. Since acoustic neuromas often occur in the cerebellopontine angle area with relatively fixed position, we combined channel attention with pixel attention in the up-sampling stage so as to make our model automatically learn different weights by adding the attention mechanism. In addition, we collected 300 MRI sequence nuclear resonance images of patients with acoustic neuromas in Tianjin Huanhu hospital for training and verification. The ablation experimental results show that the proposed method is reasonable and effective. The comparative experimental results show that the Dice and Hausdorff 95 metrics of the proposed method reach 95.74% and 1.9476 mm respectively, indicating that it is not only superior to the classical models such as UNet, PANet, PSPNet, UNet++, and DeepLabv3, but also show better performance than the newly-proposed SOTA (state-of-the-art) models such as CCNet, MANet, BiseNetv2, Swin-Unet, MedT, TransUNet, and UCTransNet.

## 1. Introduction

Acoustic neuroma is one of the most common tumors in the cerebellopontine angle area, accounting for about 85% of the tumors in this region. Although these tumors are typically non-life-threatening, postoperative morbidity can be associated with injury to the facial nerve, cochlear nerve, cerebrospinal fluid leaks, and other wound complications. Permanent facial paralysis can occur in 3 to 5% of cases, and up to 22% of patients may experience cerebrospinal fluid leaks (North et al., 2022). Fortunately, the surgical mortality rate is low, with less than 1% of cases resulting in death (McClelland et al., 2011). The main manifestation of acoustic neuroma is the thickening of the auditory nerve. Due to the limitation of bone canal, the tumor gradually grows to the cerebellopontine angle area with less resistance (Ling et al., 2016). The tumor originates from the vestibular part of the VIII pair of cranial nerves. The early lesions are small and often grow in the internal auditory canal. Neurosurgeons need to use Magnetic Resonance Imaging (MRI), which not only takes a lot of time, but also is susceptible to subjective factors. Therefore, it is of great significance to realize the automatic and accurate segmentation of acoustic neuroma. MRI has the characteristics of no bony artifacts, multi-directional and multi angle imaging, clear anatomical structure and high-level resolution for tissues. It can clearly show the size, shape, edge contour, peritumoral edema and adjacent structural changes of tumor, providing information for the preoperative diagnosis of tumor. It has become a preferred method for the examination of space occupying lesions in cerebellopontine angle (Xiaoxia et al., 2014).

At present, in the medical field, manual segmentation is mainly used in brain tumor segmentation. Manual segmentation is to manually outline the tumor area in all tumor MRI image slices. Although manual segmentation is accurate, it is time-consuming, laborious and subjective, which is not conducive to the timely diagnosis and treatment of patients. Therefore, scholars have been exploring automatic segmentation methods. In the early stage, people mainly focused on traditional segmentation methods, such as threshold segmentation (Xiaobo et al., 2019), watershed segmentation (Yongzhuo and Shuguang, 2018), region segmentation (Qiulin and Xin, 2018). There are also more complex segmentation methods based on statistical shape model [6] and graph cut (Corso et al., 2008). Despite the high speed of these segmentation methods, its result depends on the parameters specified by the user and the preprocessing of MRI images (Lingmei et al., 2020), which greatly limits its generalization ability.

With the rapid development of artificial intelligence in recent years, deep learning methods have been successfully applied to the field of medical images. Deep learning models solve the problems of poor accuracy and strong dependence on data in traditional automatic segmentation methods, such as threshold segmentation, region segmentation, and clustering segmentation, and have made great progress in medical image segmentation. AlexNet (Krizhevsky et al., 2017), VGG (Simonyan and Zisserman, 2014), GoogLeNet (Szegedy et al., 2014), ResNet (He et al., 2016), DenseNet (Huang et al., 2016), and other deep and wide network structures have been proposed one after another to learn deeper data features. UNet (Ronneberger et al., 2015) is a network structure proposed by Ronneberger et al. in 2015, which was originally applied in the field of biomedical cell segmentation. In 2019, Mumtaz et al. used a new method based on 3D fully convolutional neural networks (FCNNs; Shelhamer et al., 2016) and a 3D level set segmentation algorithm to classify and segment colon and rectal cancer. Their accuracy was 0.9378, which was 0.0755 lower than the previous accuracy of 0.8623 (Soomro et al., 2018). Cuixia et al. (2019) discussed and compared various classification models for breast tumors using deep learning in 2019 and proposed a novel method that combines deep learning features. Deep learning is also widely applied in brain tumor segmentation. Thillaikkarasi and Saravanan (2019) proposed a brain tumor segmentation algorithm using a support vector machine to extract features and CNN segmentation in 2019, resulting in an accuracy of 84%. Dong et al. (2017) used UNet to segment MRI images of brain tumors and achieved good results by splicing feature vectors of the expansion path and contraction path through skip connections. Lingmei et al. (2020) improved the UNet structure in 2020 and applied it to the segmentation of glioma magnetic resonance images. Specifically, they used an attention module on the contraction path of UNet to distribute weight to convolution layers of different sizes, promoting the utilization of spatial and contextual information. Replacing the original convolution layer with the residual compact module can extract more features and promote network convergence. In 2021, Russo et al. (2020) applied a spherical transformation preprocessing input training model, which was better than the Descartes input training model in predicting glioma tumor core segmentation and enhancing tumor category. The two models were combined to further improve prediction accuracy.

Undoubtedly, CNN represents a very promising method for image processing. However, its convolution operation has limitations, especially for samples with large texture differences, resulting in weak performance. In recent years, scholars have proposed several solutions to address this issue. For instance, Chen et al. (2014) introduced the Atrous Spatial Pyramid Pooling (ASPP) module in DeepLabv3+ (Chen et al., 2018a) after several generations of improvements (Chen et al., 2017, 2018b). The addition of ASPP into CNN enables atrous convolution to expand the vision field of the filter without increasing computational demand. Therefore, ASPP can obtain feature information of different scales without using a pooling layer, overcoming the limitations of local information loss caused by grid effect and the lack of correlation between long-distance information when using a single atrous convolution. Moreover, some studies suggest building a self-attention mechanism based on CNN features (Wang et al., 2017) as an effective means to solve the limitations of convolution operations. This method has also garnered much attention in the field of artificial intelligence. For instance, Tian et al. (2020) used channel attention in ADNet to accurately extract useful information hidden in the complex background. Huang et al. (2020) proposed the Criss-cross attention module in CCNet to capture contextual information of the complete image. Fan et al. (2020) introduced the self-attention mechanism in 2020 and proposed Multi-scale Attention Net (MA-Net).

Furthermore, Transformer has emerged as an alternative architecture designed for sequence-to-sequence prediction, and its success has been widely demonstrated in various fields such as machine translation and natural language processing (NLP; Vaswani et al., 2017; Devlin et al., 2018). In various image recognition tasks, Transformer has proven to reach or even exceed the state-of-the-art (Zheng et al., 2020; Dosovitskiy et al., 2021). For example, Chen et al. (2021) combined Transformer as a powerful encoder for medical image segmentation tasks with UNet in 2021, proposing TransUNet as a powerful alternative for medical image segmentation. Yang et al. added an attention mechanism to TransUNet (Yang and Mehrkanoon, 2022), showing that the combination of attention mechanism and TransUNet can optimize the segmentation effect. Subsequently, Valanarasu et al. proposed the MedT (Valanarasu et al., 2021) containing Local–Global (Logo) training strategy based on Transformer, which further improved the model’s performance. Cao H et al. fused high-resolution features from different scales of the encoder by skip connections, and Swin-Unet (Cao et al., 2021) was proposed to mitigate the loss of spatial information due to the pooling operation.

It is worth noting that acoustic neuromas have different shapes and may grow into the inner auditory canal, which is challenging for accurate feature extraction. We believe that the combination of ASPP, attention mechanism and Transformer can solve this challenge well. Therefore, we propose a novel model called ACP-TransUNet for accurate segmentation of acoustic neuromas, with TransUNet as the core framework. Specifically, the ASPP module is added to increase the receptive field, enabling more accurate and noticeable extraction of tumor features during the segmentation process. We also incorporate the CPAT module, which combines channel attention (Jie et al., 2019) and pixel attention (Zhao et al., 2020) to better explore channel and pixel features of acoustic neuromas while recovering the original input image size. The use of feature multiplication between attentions enhances the ability of feature representation and improves the feature propagation strategy, resulting in higher performance under the same computational load (Zhao et al., 2020; e.g., RCAN, Zhang et al., 2018; CARN, Ahn et al., 2018). By arranging the channel attention and pixel attention sequentially, we aim to improve the feature extraction capability of ACP-TransUNet.

Our main contributions are as follows:

1. Our proposed ACP TransUNet combines Transformer and CNN to capture the global and local features of the segmentation target.
2. In the down-sampling process, the ASPP module is added after the convolutional neural network to gain contextual information at multiple scales and resolutions.
3. In the up-sampling process, channel attention and pixel attention are used to improve model performance and accuracy by weighting important features.

## 2. Related works

### 2.1. TransUNet

UNet has become the most commonly used method to accurately segment lesions in medical segmentation tasks, and Transformer has also become a structural system that replaces the self-attention mechanism. TransUNet combines Transformer with UNet as a powerful alternative for medical image segmentation, possessing the advantages of both. To compensate for the loss of feature resolution due to Transformers, TransUNet adopted a hybrid CNN-Transformer architecture to exploit the detailed high-resolution spatial information of CNN features and the global context encoded by Transformers. Inspired by U-Shape, the attention features encoded by Transformers are combined with different high-resolution CNN features during upsampling to achieve precise localization. This design enables the model to preserve the advantages of Transformer and also facilitates the segmentation of medical images. On the one hand, Transformer encodes the tokenized image patches of the convolutional neural network (CNN) feature map as an input sequence for feature extraction; on the other hand, the decoder up-sampling the encoded features, and then combines them with the feature map in CNN to achieve accurate positioning (Chen et al., 2021). Currently, TransUNet and its variants have achieved great success in image segmentation. Nurçin used TransUNet for the segmentation step of the red blood cells to improve the segmentation quality of overlapping cells (Nurçin, 2022). MS-TransUNet++ (Wang et al., 2022) employed a multi-scale and flexible feature fusion scheme between different levels of encoders and decoders to achieve competitive performance in prostate MR and liver CT image segmentation. Liu et al. proposed an efficient model called TransUNet+ (Liu et al., 2022) through a redesigned skip connection, which has achieved promising results in medical image segmentation. Wang et al. proposed UCTransNet (Wang et al., 2021), which used the CTrans block to replace the skip connection in U-Net and obtained a higher segmentation effect. DS-TransUNet (Lin et al., 2022) applied swin transformer block (Liu et al., 2021) to encoder and decoder. This may be the first attempt to combine the advantages of layered Swin Transformer into both encoder and decoder of standard U-shaped architecture with the aim of improving the segmentation quality of different medical images. In TransAttUnet (Chen et al., 2021), multilevel guided attention and multiscale skip connection were co-developed to effectively improve the functionality and flexibility of the traditional U-shaped architecture. Zhao et al. proposed an automatic deep learning pipeline nn-TransUNet (Zhao et al., 2022) for cardiac MRI segmentation by combining the experimental planning of nn-UNet and the network architecture of TransUNet. EG-TransUNet (Pan et al., 2023) used progressive enhancement module, channel spatial attention, and semantic guidance attention to be able to capture object variability on different biomedical datasets. In summary, the architecture of TransUNet combines the advantages of Transformer and CNN, which is not only good for local information extraction, but also can explore long-range modeling.

### 2.2. Channel attention

**Figure 1:**
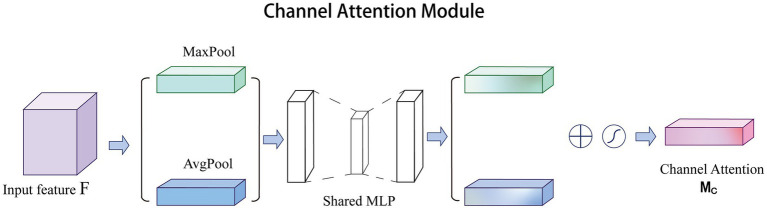
Overview of the channel attention structure.

Channel attention was first proposed in SE-Net and achieved excellent performance. In CBAM (Woo et al., 2018), channel attention has been improved significantly. Specifically, channel attention compresses the feature of spatial dimension, i.e., each two-dimensional feature map becomes a real number, which is equivalent to the pooling operation with global receptive field. The number of feature channels remains unchanged, and the module structure is shown in Figure 1. Channel attention aggregates spatial information of feature maps based on global average pooling AvgPool(F) and maximum pooling MaxPool(F) operations, generating two different spatial context descriptors: $F_{\mathrm{avg}}^{c}$ and $F_{\max}^{c}$ , representing average pool features and maximum pool features, respectively. After adding the two feature maps of the multilayer perceptron (MLP), the Sigmoid function is used to generate channel feature map, as follows in Eq. (1):

(1) $$\begin{array}{l} M_{C}\left(F\right)=\sigma \mathrm{(MLP}(\mathrm{AvgPool}(F))+\\ \mathrm{MLP(MaxPool(F)))}=\sigma \\ \left(W_{1}(W_{0}(F_{\mathrm{avg}}^{c}))+W_{1}(F_{\max}^{c}))\right) \end{array}$$

where σ represents the Sigmoid function, $W_{0}$ and $W_{1}$ represent the two convolution operations, respectively, and $F_{\mathrm{avg}}^{c}$ and $F_{\max}^{c}$ represent the average pooling and max pooling, respectively. Sigmoid function can map the result to 0–1 with the amplitude unchanged, so we can get the weight of each feature point of the input channel feature layer.

In recent years, channel attention has been widely used to solve medical challenges. Yuan et al. improved the accuracy of automatic vessel segmentation in fundus images by embedding an adaptive channel attention module to automatically rank the importance of each feature channel (Yuan et al., 2021). Du et al. applied channel attention to the automatic segmentation of early gastric cancer (EGC) to extract subtle discriminative features of EGC lesions by capturing the interdependence between channel features (Du et al., 2023). In addition, channel attention paired with other excellent attention mechanisms can also improve the quality of super-resolution reconstruction of medical images. Song et al. and Zhu et al. obtained high-quality reconstructed images for glioma MRI images and lung cancer CT images, respectively (Zhu et al., 2022; Song et al., 2023). Therefore, channel attention has great potential in the field of medical image processing.

### 2.3. Pixel attention

**Figure 2:**
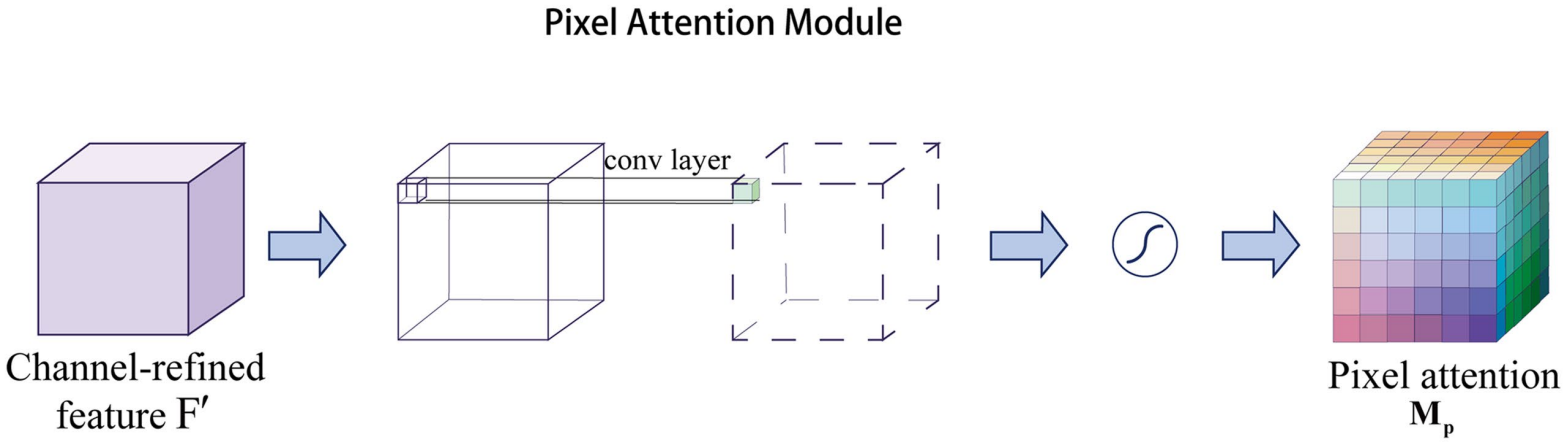
Overview of pixel attention structure.


The channel attention aims to obtain a 1D(C×1×1) vector of attentional features. In contrast, pixel attention (Zhao et al., 2020) is able to generate 3D(C×H×W) matrices as attention features. Note that C is the number of channels, and H and W are the height and width of the features, respectively. Specifically, pixel attention generates attention coefficients for all pixels of the feature map. As shown in Figure 2, pixel attention uses only 1 × 1 convolutional layers and Sigmoid functions to obtain the attention map, and then multiplies the attention map with the input features, as follows in Eq. (2):

(2) $$M_{P}\left(F^{\prime }\right)=\sigma \left(f_{\mathrm{PA}}^{1\times 1}\left(F^{\prime }\right)\right)$$

where σ represents the Sigmoid function and $f_{\mathrm{PA}}^{1\times 1}$ represents a convolution operation with the filter size of 1×1 .

Pixel attention not only reduces the number of parameters, but also eliminates unnecessary pooling operations that can lead to image smoothing (Tang et al., 2021). Relying on this advantage, pixel attention is widely used in the field of medical images for segmentation (Roy et al., 2022) and super-resolution reconstruction tasks (Rajeshwari and Shyamala, 2023).

## 3. Methods

### 3.1. Overview

**Figure 3:**
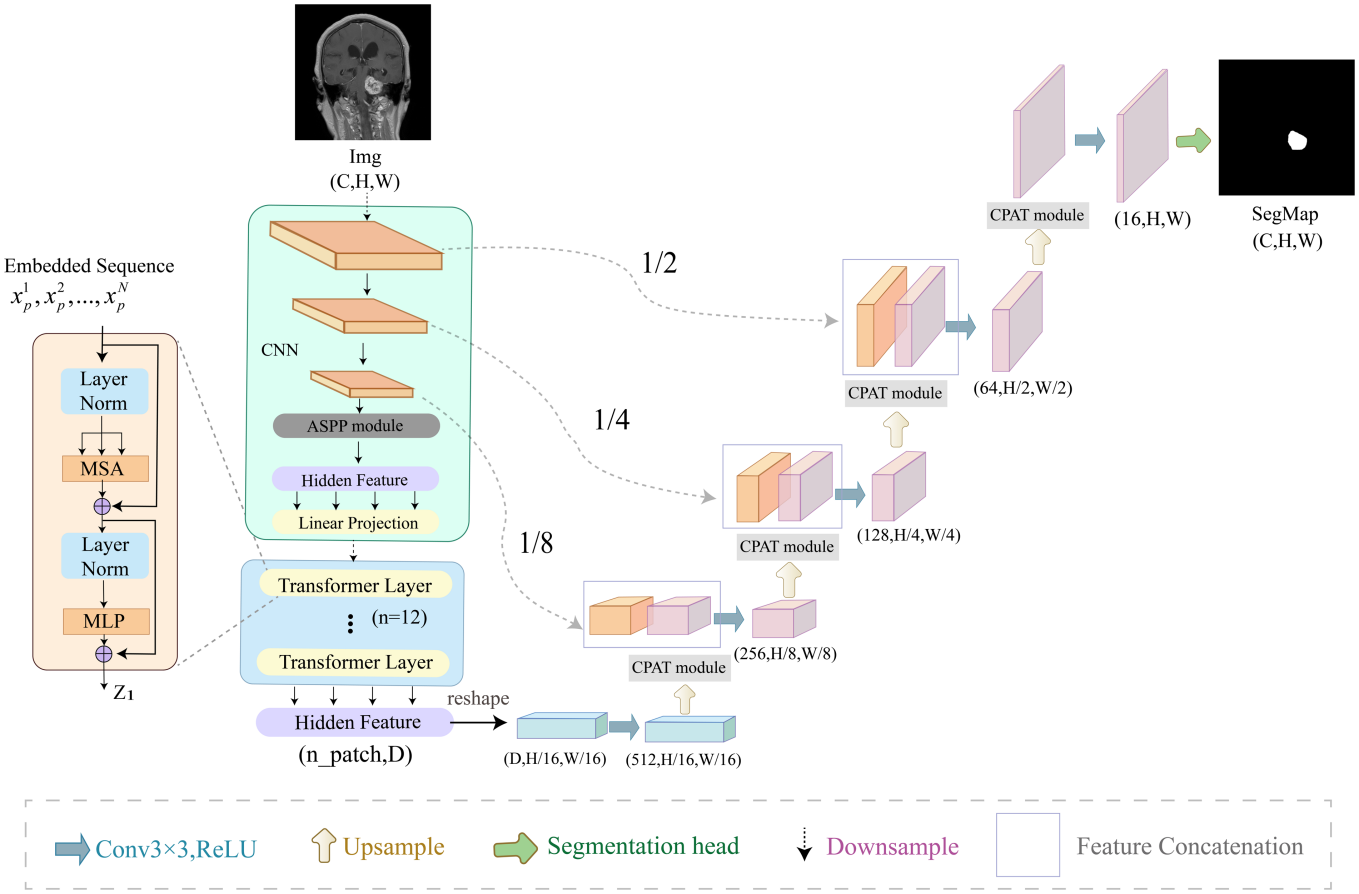
Overview of ACP-TransUNet. The input is an acoustic neuroma MRI image, and the output is the corresponding prediction map generated by ACP-TransUNet.

In this section, we describe our ACP-TransUNet with more details. The ACP-TransUNet model proposed in this paper is based on the TransUNet (Chen et al., 2021) model, and is improved and extended on the basis of the latter, as shown in Figure 3.

Given an input image with resolution H×W and C number of channels, the segmentation map is obtained by down-sampling and up-sampling. The down-sampling process consists of five parts, which are CNN, ASPP, Image Sequentialization, Patch Embedding, and Transformer Layer. The input image is first extracted by CNN layer to get the feature map. After that, the ASPP module is used to increase the receptive field to obtain a feature map with different scales. Then, Hidden Feature and Linear Projection reshape the feature map into N flattened 2D patches for Image Sequentialization, with each patch of size P×P , $N=\frac{H\prime W\prime }{P^{2}}$ , $H^{\prime }$ and $W^{\prime }$ being the length and width of each feature map. In order to encode the spatial information of the patches, we add positional embedding to the patch embedding to preserve the positional information, as follows in Eq. (3):

(3) $$Z_{0}=\left[x_{p}^{1}{\mathrm{E; x}}_{p}^{2}\mathrm{E;}\ldots \;;\;x_{p}^{N}E\right]+E_{p}$$

where $E\in {\mathbb{R}}^{\left(P^{2}.C\right)\times D}$ represents the patch embedding projection, $x_{p}$ represents the vectorized patch, and $E_{P}\in {\mathbb{R}}^{N\times D}$ represents the position embedding.

The Transformer (Vaswani et al., 2017) layer is added at the end of the down-sampling to obtain the global features, which consists of Multi-head Attention (MSA) and Multi-layer Perceptron (MLP) as shown in Eqs. (4) and (5):


(4) $$Z_{n}^{\prime }=\mathrm{MSA}\left(\mathrm{LN}\left(z_{n-1}\right)\right)+z_{n-1}$$

(5) $$Z_{n}=\mathrm{MLP}\left(\mathrm{LN}\left(z_{n}^{\prime }\right)\right)+z_{n}^{\prime }$$

where LN(·) denotes the layer normalization operator and $z_{n}$ is the encoded image representation.

In the up-sampling process, we added CPAT modules in each layer to weight the important features in recovering the image size to improve the performance and accuracy of the model.

### 3.2. ASPP module

**Figure 4:**
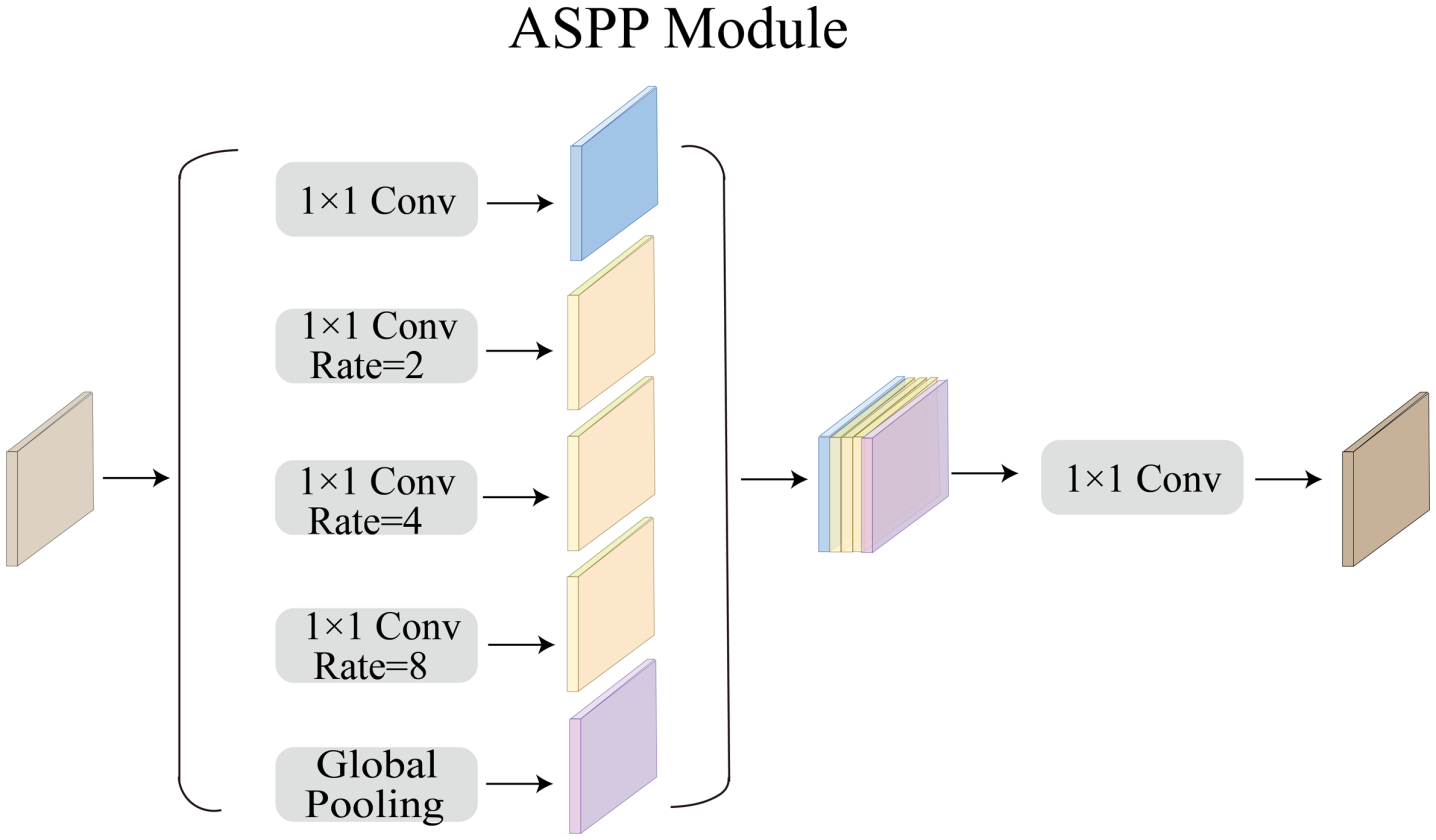
Overview of ASPP Module.

Acoustic neuromas vary in shape. Some are irregular in shape and grow into the inner auditory canal, while some have clear boundary. Therefore, we need a larger receptive field to extract the feature of acoustic neuromas. The ordinary convolution structure cannot fully extract features, so in this paper we choose to use ASPP module to strengthen the ability of the model to segment objects at different scales. As shown in Figure 4, in this paper, ASPP module is equipped in the last layer of CNN, with dilation rate set to 2, 4, 8. The rate of atrous convolution is based on the ordinary convolution, and the interval between adjacent weights is rate−1 . The rate of ordinary convolution is defaulted to 1, so the actual size of atrous convolution is k+(k−1)(rate−1) , in which k is the size of the original convolution kernel. ASPP overcomes the shortcomings of local information loss and lack of correlation in remote information caused by grid effect when using single atrous convolution, making it possible to obtain different scale feature information without using pooling layer.

### 3.3. CPAT module

**Figure 5:**
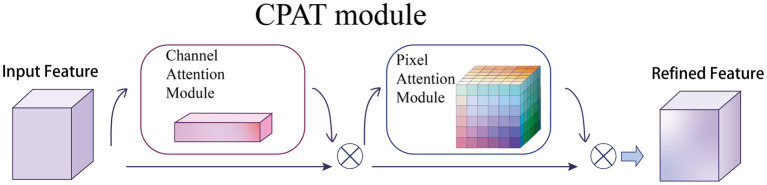
Overview of CPAT structure. This module has two submodules: channels and pixels, where ⊗ denotes element-wise multiplication. The intermediate feature map is adaptively refined through our module (CPAT).

Given an intermediate feature map $F\in {\mathbb{R}}^{C\times H\times W}$ as input, CPAT module sequentially infers a 1D channel attention map $M_{c}\in {\mathbb{R}}^{C\times 1\times 1}$ and a 3D pixel attention map $M_{P}\in {\mathbb{R}}^{C\times H\times W}$ as illustrated in Figure 5. For the arrangement of attention modules, we found through experiments that the result is better when using two sequential attentions than using one attention, which will be discussed in the ablation experiments, as shown in Eqs. (6) and (7):

(6) $$F^{\prime }=M_{c}\left(F\right)⊗F$$

(7) $$F^{\prime \prime }=M_{P}\left(F^{\prime }\right)⊗F^{\prime }$$

where $F^{\prime }$ denotes the feature map obtained by channel attention, $F^{\prime \prime }$ denotes the feature map obtained by pixel attention, and ⊗ denotes element multiplication.

## 4. Experimental results

In this section, we introduce the details of the experimental data and results. In order to verify whether ACP-TransUNet can effectively and accurately segment acoustic neuromas, we first performed comparative experiments and ablation experiments on all test sets (including coronal view, sagittal view, and transverse view). To test the accuracy of the model’s segmentation effect in a single view, we also conducted multi-view evaluation, performing a comparison experiment and ablation experiment on the three views separately. The results are discussed in detail below. Among them, ACP-TransUNet achieves 95.74% Dice Similarity Coefficient on the test set, and Hausdorff 95 reaches 1.9476 mm, which are superior than other models.

### 4.1. Dataset

We selected MRI images of sagittal view, coronal view and transverse view of patients with cerebellopontine angle (CPA) acoustic neuroma diagnosed by experts in Tianjin Huanhu Hospital from January 2019 to January 2022, with all the patients signing informed consent. The scanning equipment we used was Siemens Skyra 3.0 T MRI scanner, which could collect magnetic resonance images of multiple sequences. However, compared with other sequences, T1WI-SE could better distinguish the lesion and its surrounding adjacent tissues. Therefore, this paper adopts contrast - enhanced fast low-angle shot 2-dimensional sequence (T1_fl2d) with Gd-GDPA. Scanning parameters are as follows: slice thickness is 5 mm; slice interval, 1.5 mm; echo time (TE), 2.46 ms; repetition time (TR), 220 ms. After screening, a total of 300 magnetic resonance images of acoustic neuromas were selected in this paper, in which the ratio of training set, verification set and test set is 8: 1: 1 and each part has no cross.

### 4.2. Preprocessing

**Figure 6:**
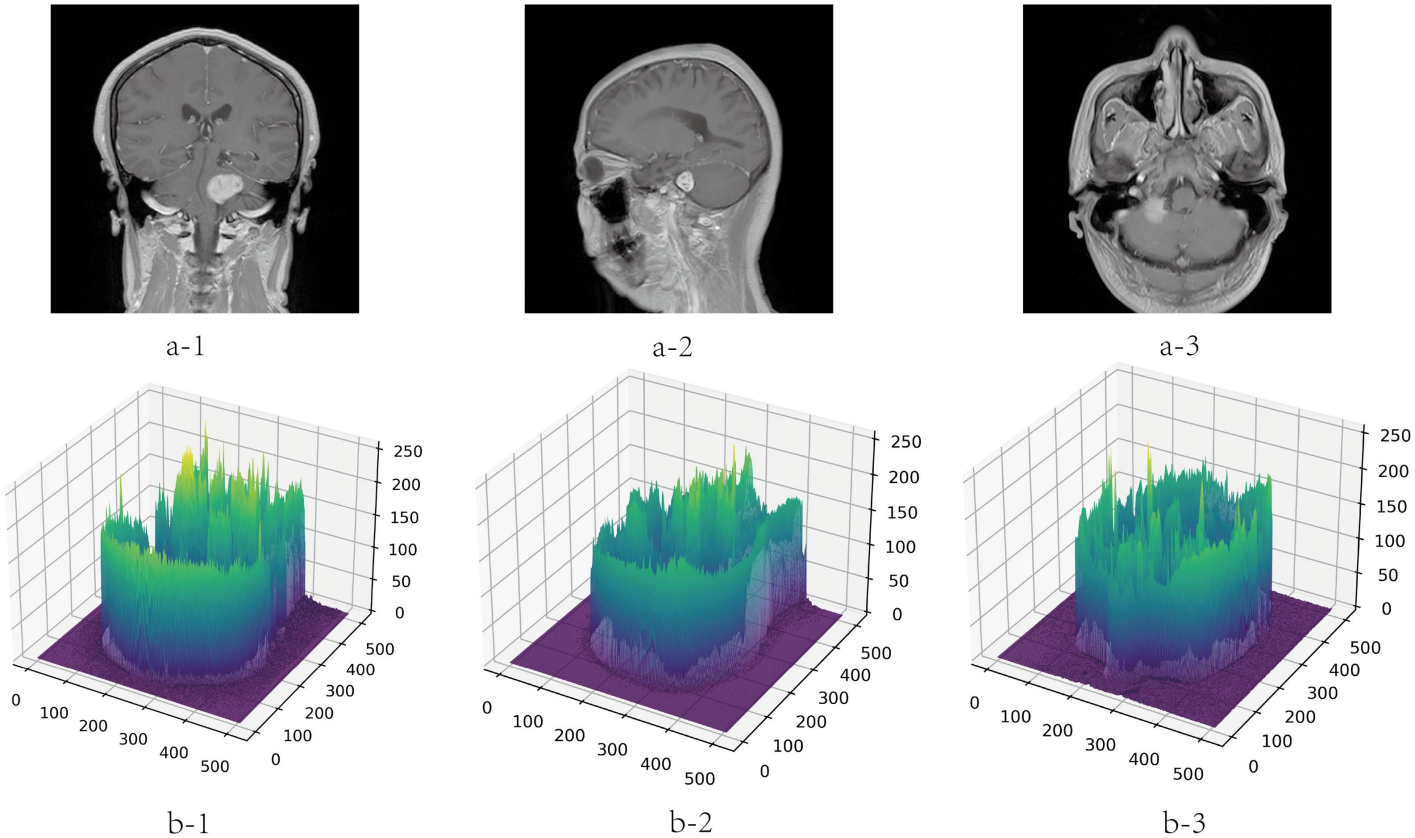
Gray visualization of MRI images in three directions. a-1, a-2, and a-3 represent coronal, sagittal and transverse MRI images, respectively. b-1, b-2, and b-3 are three-dimensional gray-scale visualization images of nuclear magnetic resonance, which represent the corresponding directions. The x-axis and y-axis represent the length and width of the image respectively, and the value range is [0, 512]. The z-axis represents the gray value distribution of the image, and the value range is [0, 255].

To avoid the deviation of the experimental results caused by the inconsistent data format, the training, verification and test MRI images in this paper are all set to the same format. Because the dataset is small, to improve the generalization ability of the model, the images are subjected to data augmentation processing such as inversion and flipping. In order to save training resources, the images are set to 512×512 pixels. The gray value visualization of the MRI image is shown in Figure 6.

### 4.3. Experimental setup

In the experiment, the framework we used was Pytorch, and batchsize was set to 4. All networks trained 100 epochs on Nvidia Tesla V100 GPU. Specifically, we used a pre-training model (R50 + ViT-B_16) that was trained on the ImageNet21k dataset. The pre-training model can be found at the following link: https://console.cloud.google.com/storage/vit_models/. In addition, we use the Adam optimizer (Kingma and Ba, 2014) to optimize, the initial learning rate is $10^{-4}$ , and use the StepLR mechanism to set the learning rate attenuation according to epoch. The StepLR mechanism is a way to adjust the learning rate during training in machine learning. It reduces the learning rate by a certain factor after a fixed number of epochs or iterations. We set the “step_size” parameter to 7 and the “gamma” parameter to 0.1, which means that the learning rate was reduced by a factor of 0.1 every 7 epochs. By gradually reducing the learning rate, we aimed to improve the convergence of the model and prevent overfitting.

### 4.4. Evaluation metrics

In order to objectively evaluate the results of different models, this paper uses the Dice Similarity Coefficient (Mehta, 2015; Liu et al., 2020) and Hausdorff 95 (Huttenlocher et al., 1993; Beauchemin et al., 1998) as representative segmentation performance indicators, which measure the similarity and maximum mismatch between the segmentation result and the labeling result, respectively. These metrics are widely used in medical image segmentation studies and have been shown to be effective in evaluating segmentation performance.

### 4.5. Comparative experiment

**Figure 7:**
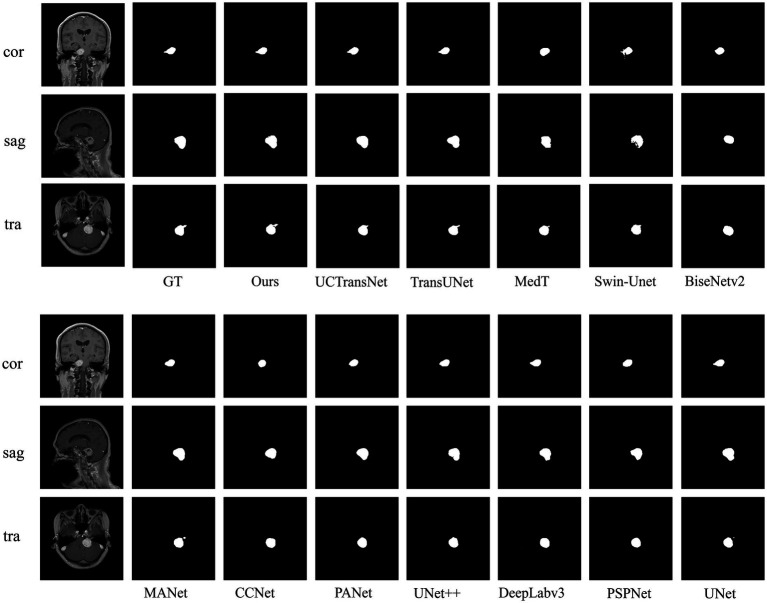
Examples of predictions for each network on acoustic neuromas in comparative experiments.

To verify the validity of the proposed model, we compared several classical networks such as PANet (Liu et al., 2018), PSPNet (Zhao et al., 2016), UNet++ (Zhou et al., 2018), and DeeplabV3 (Chen et al., 2018a), as well as some emerging networks such as CCNet (Huang et al., 2020), MANet (Fan et al., 2020), BiseNetv2 (Yu et al., 2021), Swin-Unet (Cao et al., 2021), MedT (Valanarasu et al., 2021), TransUNet (Chen et al., 2021), and UCTransNet (Wang et al., 2021), which have shown great performance on segmentation tasks in recent years. Table 1 summarizes the comparison results between our scheme and these representative networks. For each model, we visualized the segmentation effect in the coronal (cor), sagittal (sag), and transverse (tra) views, and the results are shown in Figure 7.

**Table 1:** Results of comparative experiment.

Model	Dice (%)	Hausdorff 95 (mm)
UNet (2015)	94.65	4.4982
PSPNet (2016)	93.11	3.2145
DeepLabv3 (2017)	93.46	4.4438
UNet++ (2018)	94.66	3.7744
PANet (2018)	93.88	4.3399
CCNet (2020)	85.32	5.2548
MANet (2020)	94.95	3.7483
BiseNetv2 (2021)	89.86	5.6903
Swin-Unet (2021)	91.46	6.3458
MedT (2021)	93.26	4.7794
TransUNet (2021)	95.02	4.0037
UCTransNet (2022)	95.06	4.0746
Ours	**95.74**	**1.9476**

Bold font is the best data for each column.

The results show that ACP-TransUNet achieved the best performance on the test set, with a Dice value of 95.74% and a Hausdorff 95 value of 1.9476 mm. Compared with the original UNet network proposed by Ronneberger et al. (2015), ACP-TransUNet achieved improvements of 1.09% and 2.5506 mm in Dice and Hausdorff 95, respectively.

In the comparative experiments, our scheme achieved optimal Dice and Hausdorff 95 values, outperforming other network models. Specifically, our scheme improved Dice by 2.63% (PSPNet), 2.28% (DeepLabv3), 1.08% (UNet++), 1.86% (PANet), 10.42% (CCNet), 0.79% (MANet), 5.88% (BiseNetv2), 4.28% (Swin-Unet), 2.48% (MedT), 0.72% (TransUNet), and 0.68% (UCTransNet), respectively. Hausdorff 95 was increased by 1.2669 mm (PSPNet), 2.4962 mm (DeepLabv3), 1.8268 mm (UNet++), 2.3923 mm (PANet), 3.3072 mm (CCNet), 1.8007 mm (MANet), 3.7427 mm (BiseNetv2), 4.3982 mm (Swin-Unet), 2.8318 mm (MedT), 2.0561 mm (TransUNet), and 2.127 mm (UCTransNet), respectively. The corresponding segmentation effect in Figure 7 demonstrates the superior performance of ACP-TransUNet.

In comparison experiments, for some regular acoustic neuromas, such as the tumor shown in the sagittal view, it can be seen that the selected networks can achieve basic segmentation of the tumor except for BiseNetv2 and MedT. However, comparing the internal filling and boundary of the segmentation map, only ACP-TransUNet is closest to Ground Truth; for the part that shows irregular shape and grows into the internal auditory canal, as shown in the coronal view, PSPNet, PANet, CCNet, BiseNetv2 and MedT cannot well segment some tumors growing in the internal auditory canal. Although UNet++ and MANet could segment the tumors in the internal auditory tract, the segmentation results were inferior to the rest of the networks. DeepLabv3, UNet and TransUNet performed comparably to ACP-TransUNet for segmenting the tumors in the internal auditory tract, but UCTransNet and ACP-TransUNet outperformed the rest of the models in terms of edge detail. However, in the transverse (tra), only TransUNet and ACP-TransUNet can well segment the acoustic neuroma. We noticed that the models containing Transformer structures (such as MedT, Swin-Unet, TransNet, and UCTransNet) were deficient in processing edge details, which may be explained by the limited Transformer localization ability caused by insufficient low-level details. After adding the CPAT module and ASPP module, the segmentation map edge contours have been greatly improved.

### 4.6. Multi-view evaluation

To further verify the effectiveness of the model, we conduct comparative experiments and ablation experiments on the segmentation effects of the coronal, sagittal and transverse views in the test set, respectively. The results of the multi-view evaluation in the comparative experiments are shown in Table 2.

**Table 2:** Comparative experiment from multiple perspectives.

Model	Dice (%)			Hausdorff 95 (mm)		
	cor	sag	tra	cor	sag	tra
UNet (2015)	94.16	94.76	94.94	7.6671	1.6476	3.8948
PSPNet (2016)	92.3	92.11	94.13	3.454	2.6466	3.4861
DeepLabv3 (2017)	92.43	93.62	94.11	7.23	1.8961	3.9505
UNet++ (2018)	93.12	94.94	95.6	7.0516	1.4254	2.6112
PANet (2018)	93.14	94.03	94.33	7.4731	1.5437	3.7234
CCNet (2020)	90.9	90.15	93.76	8.0307	2.7355	4.7462
MANet (2020)	93.83	94.52	95.91	6.8507	2.4254	**1.8365**
BiseNetv2 (2021)	89.84	84.93	92.07	8.3099	4.3865	4.2442
Swin-Unet (2021)	88.9	89.93	93.8	10.3311	4.7801	3.7695
MedT (2021)	91.52	92.32	94.89	8.2466	3.1183	2.8071
TransUNet (2021)	94.31	94.34	95.81	6.7094	2.3035	2.8282
UCTransNet (2022)	94.4	94.01	95.99	7.5424	2.6062	1.9285
Ours	**94.88**	**95.45**	**96.45**	**2.541**	**1.4056**	1.902

Bold font is the best data for each column, and the coronal, sagittal, and transverse views are represented by cor, sag, and tra, respectively.

It can be seen that although the Hausdorff 95 is not as good as MANet in the transverse view, our model is generally better than other models through the evaluation of dice and Hausdorff 95 values. Dice values of the coronal view, sagittal view and transverse view reached 94.88, 95.45 and 96.45% respectively; and the Hausdorff 95 values reached 2.541 mm, 1.4056 mm and 1.902 mm, respectively.

### 4.7. Ablation experiment

To demonstrate the efficacy of the incorporation module, we performed two groups of ablation experiments based on the principle of “fixing two items and changing one item.”

#### 4.7.1. Ablation experiment of attention module

We examine four different experimental configurations to verify the efficacy of adding attention modules, i.e., TransUNet with ASPP (TransUNet+ASPP) as the baseline, and further with channel attention (TransUNet+ASPP+C), pixel attention (TransUNet+ASPP+P), and CPAT module (TransUNet+ASPP+CPAT). Tables 3, 4 show the segmentation results for the overall and multiple views, respectively.

**Table 3:** Results of ablation experiments with attentional module.

Model	Dice (%)	Hausdorff 95 (mm)
TransUNet+ASPP	95.18	4.2574
TransUNet+ASPP+C	95.23	3.7512
TransUNet+ASPP+P	95.52	2.4821
TransUNet+ASPP+CPAT	**95.74**	**1.9476**

Bold font is the best data for each column.

**Table 4:** Results of ablation experiments with attentional module from multiple perspectives.

Model	Dice (%)			Hausdorff 95 (mm)		
	cor	sag	tra	cor	sag	tra
TransUNet+ASPP	94.18	94.85	95.75	6.2425	1.9845	3.8742
TransUNet+ASPP+C	94.24	94.98	95.91	5.9475	1.4863	2.8431
TransUNet+ASPP+P	94.66	95.05	96.34	5.7424	1.8574	1.9527
TransUNet+ASPP+CPAT	**94.88**	**95.45**	**96.45**	**2.541**	**1.4056**	**1.902**

Bold font is the best data for each column, and the coronal, sagittal, and transverse views are represented by cor, sag, and tra, respectively.

From Tables 3, 4, we have some observations as follows.

1. When we added channel attention to “TransUNet+ASPP,” not only the Dice and Hausdorff 95 of “TransUNet+ASPP+C” in Table 3 improved by 0.05% and 0.5062 mm, respectively, but also the experimental results of multiple views in Table 4 were better than those of “TransUNet+ASPP”， which proves the effectiveness of adding channel attention.
2. When we added pixel attention to “TransUNet+ASPP,” the Dice and Hausdorff 95 of “TransUNet+ASPP+P” in Table 3 were 95.52% and 2.4821 mm, respectively, and the experimental results in Table 4 were also improved significantly, thereby proving that the addition of pixel attention is effective.
3. The results of “TransUNet+ASPP+CPAT” in Tables 3, 4 are significantly better than those of “TransUNet+ASPP+C” and “TransUNet+ASPP+P,” demonstrating that the sequential connection of channel attention and pixel attention is better than using either attention module.

#### 4.7.2. Ablation experiment of ASPP module

To demonstrate the efficacy of the ASPP module, two different experimental configurations were studied, i.e., TransUNet with CPAT (TransUNet +CPAT) as a baseline and further addition of the ASPP module (TransUNet +CPAT+ASPP). Tables 5, 6 show the segmentation results for the overall and multiple views, respectively. As can be seen from Table 5, the addition of the ASPP module improves the “TransUNet+CPAT+ASPP” Dice and Hausdorff 95 by 0.2% and 1.5166 mm, respectively. In addition, according to Table 6, Hausdorff 95 with “TransUNet +CPAT+ASPP” is excellent in other views, although it is lower than “TransUNet +CPAT” in the transverse view. The above results prove the efficiency of ASPP module.

**Table 5:** Results of ablation experiments with ASPP module.

Model	Dice (%)	Hausdorff 95 (mm)
TransUNet +CPAT	95.54	3.4642
TransUNet +CPAT+ASPP	**95.74**	**1.9476**

Bold font is the best data for each column.

**Table 6:** Results of ablation experiments with ASPP module from multiple perspectives.

Model	Dice (%)			Hausdorff 95 (mm)		
	cor	sag	tra	cor	sag	tra
TransUNet +CPAT	94.68	95.34	96.23	7.0574	1.4682	**1.8472**
TransUNet +CPAT+ASPP	**94.88**	**95.45**	**96.45**	**2.541**	**1.4056**	1.902

Bold font is the best data for each column, and the coronal, sagittal, and transverse views are represented by cor, sag, and tra, respectively.

## 5. Discussion

At present, the results of Dice and Hausdorff distance of our model in acoustic neuroma segmentation have reached our expectations. Given the fact that acoustic neuromas vary in shape--some with irregular shape and growing into the inner auditory canal, while some with clear boundary, we need a larger receptive field to extract the feature of acoustic neuromas. As ordinary convolution structure cannot fully extract features, we added the ASPP module. Furthermore, since acoustic neuromas often occur in the cerebellopontine angle area with relatively fixed position, we intended to make our model automatically learn the weights at different scales by adding the attention mechanism. Therefore, we added the channel attention and pixel attention in the up-sampling, so that the channel information and pixel information are combined to better explore the channel characteristics and pixel characteristics while restoring the original input image size. In the comparison experiments, we can see that most of the networks with the added Transformer structure achieve good results in segmentation of acoustic neuromas, for example, the Dice value of these networks is almost equal to that of ACP-TransUNet. However, Hausdorff 95 cannot be comparable to ACP-TransUNet. which is due to Transformer’s inadequacy to capture low-level details and its limited positioning ability. Given that, we combined ASPP and attention mechanism to make up for this deficiency. In the ablation experiment, it is observed that the segmentation performance of the model becomes better and better with the addition of ASPP and CPAT modules, proving the effectiveness of our choice to add the modules.

However, there are still problems existing in the current work. For example, in the multi-view evaluation, we did not achieve desirable segmentation results in the transverse view. The Hausdorff 95 value of our model in the transverse view is 1.902 mm. That figure is inferior to the MANet, which reached 1.8365 mm in the comparison experimental. The reasons we believe are of two aspects. First, it could be explained by the relatively low importance of channel weight in the down-sampling of acoustic neuromas in the transverse view direction. But the addition of pixel attention could make all the pixels of the feature map generate attention coefficient, which makes up for the disadvantage of using channel attention alone. Second, although the addition of ASPP module would increase the receptive field, making each convolution output contain a large range of information, the information of smaller tumors in the transverse view could be lost. Given that, in our future work, we will gradually increase the dataset and study the performance changes when increasing or decreasing the single direction module. In addition, our current research task is to achieve accurate segmentation of acoustic neuromas. We hope that the application of ACP-TransUNet will not be limited to acoustic neuromas, so its effectiveness in segmenting other medical images will also be the focus of our future experimental research.

In our research work, the improvement of the accuracy of acoustic neuroma segmentation means that we need to abandon some indicators in some aspects. We have considered trade-offs in these issues. First, the addition of ASPP module, attention mechanism and deeper transformer layer means longer training time and larger model parameters. We believe that the medical segmentation task is different from other segmentation tasks that pursue timeliness (such as face segmentation). Between lightweight and precision, we prefer the latter. Second, since Transformer lacks the inductive bias of convolution, it requires more sample size than CNN. Transformer needs to learn this kind of information from a large amount of data. Considering the precious resources and insufficient data support of current medical images, instead of choosing to train from scratch, we resort to pre-trained models to achieve the same or even better performance than CNN. In the future, we will conduct research for Transformer on small-scale datasets.

## 6. Conclusion

In this paper, we proposed a novel model named ACP-TransUNet based on the improved TransUNet structure, with all the data on the basis of MRI images. Through deep learning, we realized the automatic and accurate segmentation of acoustic neuromas in the cerebellopontine angle region. Dice and Hausdorff 95 reached 95.74% and 1.9476 mm respectively, and the dividing boundary was closer to the gold standard. The overall effect of segmentation was significantly improved, which was valuable for clinical application and auxiliary physician diagnosis. With decreased intervention of human factors, we greatly improved the diagnostic efficiency and reliability. In addition, the ASPP module was introduced into ACP-TransUNet, which not only increases the receptive field and obtains multi-scale and multi-resolution background information, but also makes the features contained in the sequence of the imported Transformer more accurate and significant. The CPAT module with sequential channel attention and pixel attention is added to the upsampling process so that channel information and pixel information are combined to improve model performance and accuracy by weighting important features. The experimental results show that our model can effectively segment acoustic neuroma. Compared with other methods, the proposed method has different degrees of performance improvement in the segmentation of acoustic neuroma.

## Data availability statement

The original contributions presented in the study are included in the article/supplementary material, further inquiries can be directed to the corresponding author.

## Ethics statement

Ethical review and approval was not required for the study on human participants in accordance with the local legislation and institutional requirements. The patients/participants provided their written informed consent to participate in this study.

## Author contributions

ZZ and HB: conceptualization. HB: methodology, formal analysis, supervision, and funding acquisition. ZZ: software, data, writing original draft preparation, and visualization. ZZ, HB, and QM: validation. JL and XZ: investigation. YY: resources. HB, QM, and CZ: writing review and editing. QM: project administration. All authors contributed to the article and approved the submitted version.

## Funding

This work was supported in part by National Natural Science Foundation of China (61201106) and Tianjin Research Innovation Project for Postgraduate Students (2022SKY126).

## Conflict of interest

The authors declare that the research was conducted in the absence of any commercial or financial relationships that could be construed as a potential conflict of interest.

## Publisher’s note

All claims expressed in this article are solely those of the authors and do not necessarily represent those of their affiliated organizations, or those of the publisher, the editors and the reviewers. Any product that may be evaluated in this article, or claim that may be made by its manufacturer, is not guaranteed or endorsed by the publisher.
